# Models of buffering of dosage imbalances in protein complexes

**DOI:** 10.1186/s13062-015-0063-8

**Published:** 2015-08-15

**Authors:** Reiner A. Veitia, James A. Birchler

**Affiliations:** Institut Jacques Monod, 15 rue Hélène Brion, 75013 Paris, France; Université Paris Diderot, Paris, France; University of Missouri, Division of Biological Sciences, Columbia, MO 65211 USA

**Keywords:** Aneuploidy, Haploinsufficiency, Trisomy, Gene deletion, Gene duplication, Polyploidy

## Abstract

**Background:**

Stoichiometric imbalances in macromolecular complexes can lead to altered function. Such imbalances stem from under- or over-expression of a subunit of a complex consequent to a deletion, duplication or regulatory mutation of an allele encoding the relevant protein. In some cases, the phenotypic perturbations induced by such alterations can be subtle or be lacking because nonlinearities in the process of protein complex assembly can provide some degree of buffering.

**Results:**

We explore with biochemical models of increasing plausibility how buffering can be elicited. Specifically, we analyze the formation of a dimer AB and show that there are particular sets of parameters so that decreasing/increasing the input amount of either A or B translates into a non proportional (buffered) change of AB. The buffer effect also appears in higher-order structures provided that there are intermediate subcomplexes in the assembly process.

**Conclusions:**

We highlight the importance of protein degradation and/or conformational inactivation for buffering to appear. The models sketched here have experimental support but can be further tested with existing biological resources.

**Reviewers:**

This article was reviewed by Eugene Koonin, Berend Snel and Csaba Pal.

## Background

Stoichiometric change in a macromolecular complex can induce altered function. Consistently, subunits of such complexes tend to have similar expression levels, most of the time assessed at the transcriptional level [1, 2]. This property stems from principles of cellular economy, but also, as explained below, as a requirement to avoid the formation of inactive sub-complexes when there is an imbalance in the concentration of one or several subunits. For instance, dosage effects within macromolecular complexes or even between transcription factors (TFs) and their cognate *cis-*regulatory target sequences explains, at least in part, the extensive retention of paralogs in polyploids [3, 4].

**Fig. 1:**
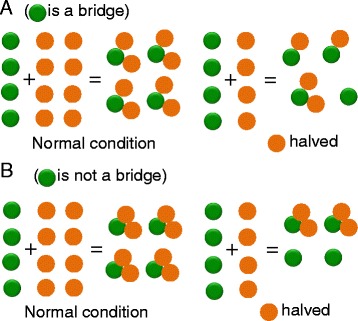
Complex topology and dosage sensitivity. **a** Case of a trimer in which the green subunit is a bridge. Halving of the latter leads to halving trimer output. However, halving the amounts of the orange subunits can lead to as low as 25 % of trimer. Overexpression of the orange subunits can be inconsequential from the perspective of the trimer output but entails a futile cost, whereas overexpression of the green ones leads to a titration effect (formation of dimeric subcomplexes), which reduces trimer output. **b** When the orange  subunits can, for instance, preassemble, halving their amount translates into a proportional decrease of trimer and the increase of the green  ones does not alter the amount of trimer. This shows how the topology of the complex and not only its composition modulate dosage sensitivity

To gain insight into the biochemical bases of the effects of stoichiometric imbalances, we explore the consequences of under- or over-expression of a subunit of a complex due, for instance, to a deletion, duplication or regulatory mutation of an allele encoding the relevant protein in a diploid organism. In such cases there is an imbalance that increases the concentration of unpartnered subunits. The deficit of one binding partner leaves the remaining partners in (relative) excess. When this situation engenders a dominant phenotype we speak of haploinsufficiency (HI). The molecular bases of HI can be diverse, depending on the systems under consideration [5]. In the case of macromolecular complexes, the explanation of some cases of HI is almost intuitive. For instance, if the complex is a trimer that contains 2 molecules of A and one molecule of B, the effect of a decrease in the concentration of A is probably more dramatic than for B, as shown in Fig. 1. However, as also shown in this figure, the result depends on the topology of the complexes [5, 6]. These examples epitomize why the expression levels of the subunits of a complex evolve to be co-regulated. Although most of what follows focuses on situations arising in diploid organisms, it is worth saying that the dosage balance principles hold also for prokaryotes. Indeed, a systematically analysis of macromolecular complexes from *E. coli* has shown that more than half of the relevant molecules are synthesized at levels (counted as molecules per generation) very well correlated with their stoichiometry within the complexes [7]. This proportional synthesis out of a polycistron relies on translational tuning [7, 8]. Not surprisingly, similar results have been found for stable complexes from *S. cerevisiae* [7].

Monomers in absolute or relative excess can be either not in their final conformation, misfolded or at least expose interfaces, which mediate protein-protein interactions, that should normally be hidden within the complex. Such interfaces tend to be rich in hydrophobic residues that contribute to protein-protein association [9]. Chaperons may protect the exposed interfaces and hide them from the aqueous surroundings. Monomers in excess can also expose normally buried degradation signals, which can lead to their efficient degradation [10]. In other cases, subunits in excess may expose hydrophobic or aggregation-prone regions that may lead to their aggregation and accumulation. Hence HI of subunits of macromolecular complexes can be due to plain insufficiency of the complex *per se* but can also lead to abnormal molecular interactions resulting in misfolding or aggregation. This can obviously be the case for overexpressed isolated subunits [11, 12].

It is well known that genetic dominance is a matter of non-linearity in the relationship between the genotype and the phenotype [5, 13]. Dosage imbalances can be expressed as dominant traits at the phenotypic level as illustrated in general by aneuploidy [14]. In some cases, the phenotypes due to gene deletions, duplications or misregulation can be subtle or lacking because nonlinearities in the process of complex assembly can provide some degree of buffering. In line with this, a previous analysis of the formation of a heterodimer AB, has shown that halving the input amounts/concentrations of A or B (*i.e.* A0 and B0, provided that they are equal in normal conditions) does not always translate into halving the amount of AB. This analysis was based on a dimerization reaction involving similar initial “full blown” concentrations of A and B as if the reaction was taking place in a test-tube. Interestingly, there is a particular value of the dissociation constant so that halving A0 (or B0) translates into the production of 59 % of dimer AB. This particular dissociation constant is of the order of A0/4 (or B0/4). This behavior was referred to as the buffer effect of equilibrium and has a similar explanation as a trivial chemical buffer [15]. As shown below, the gain of almost 10 % of AB may be particularly important in cooperative systems where a small change of effector concentration can elicit huge changes in the response [5, 16]. This buffer effect suggests that the association constant and the concentration of the partners can be tuned by selection so as to make the system robust against halving the input of one of its components. However, taking advantage of this buffer effect implies the existence of unused free monomers. Interestingly, increasing the input concentration of either A or B (1.5 times as in a heterozygous gene duplication) for the same dissociation constant translates into 138 % of AB with respect to normal conditions (instead of 150 %). Thus, the buffer effect works in both directions (*i.e.* gene-dosage increase or decrease) and also appears in higher order structures provided that there are subcomplexes in equilibrium [15]. Notice that a maximum degree of buffering of the amount of AB is a focal effect and does not imply any kind of notion of ‘optimality’. Optimal functioning should be defined from the perspective of a “wild-type” (WT) individual in a particular environment and cannot be reduced to the amount of AB in isolation. Below, we explore the potential existence of buffering effects in situations of increasing plausibility, extending our previous views [17].

## Results and discussion

### The effect of protein degradation and/or conformational inactivation on buffering

**Fig. 2:**
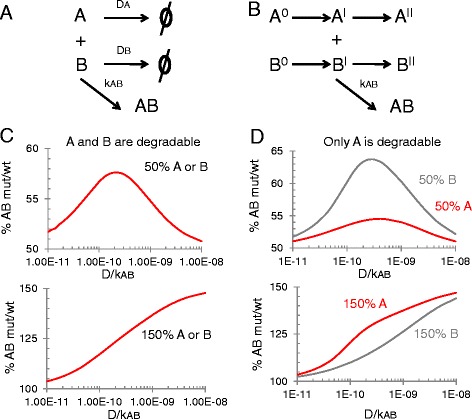
Buffer effects in the assembly of a heterodimer. **a** The monomers A and B are involved in competing reactions: their degradation or their dimerization. **b** Alternative scenario in which both A and B have a preferential conformation to interact with each other (*i.e.* A^I^ and B^I^). Conformations A^II^ and B^II^ do not lead to dimers. Note that the parameters of synthesis and degradation encapsulate information on both mRNA and protein in this simplified model, but we assume that no buffering occurs at the transcriptional level. **c** Buffering response of heterodimer AB formation to changing the input concentration of one monomer. As mentioned in the text, here we consider for simplicity that A and B are synthesized in a very short time scale compared to the rest of the reactions. So we deal with input concentrations and not with parameters of synthesis (as will be the case in Fig. 3). The ordinates represent the % of AB when either A0 or B0 are changed (0.5X or 1.5X, "mutated" condition) with respect to A0 = B0 ("wild-type", wt). The results were obtained with the biochemical simulator GEPASI, which solves numerically the chemical and the underlying differential equations [40]. If normally A0 = B0 = 1nM, DA = DB (here called D) and kAB > > D, at a specific D/kAB value, halving the input amount of either monomer (upper panel) leads to >57 % of dimer in such (rather artificial) conditions of irreversibility. Operating at the same D/kAB value leads to 123 % of AB output when A0 or B0 are increased by 150 %. **d** Response of heterodimer AB formation to changing the input concentration of one monomer (when one of them can be degraded and the other not). In this case A0 = B0 = 1nM, DA = 0.01 min^−1^ and DB = 0 min^−1^

We will now analyze the assembly of a protein complex, namely the dimer AB, in irreversible conditions, when the amount of one subunit is decreased or increased, according to the reaction scheme represented in Fig. 2a. We focus first on this rather artificial system involving initial fixed amounts of A and B in order to more easily dissect the impact of the competing reactions, namely dimerization and degradation, on the level of buffering. This model would apply, for instance, to a situation in which there is a rapid burst of production of A and B and in which AB is more stable than the free monomers, which might expose degradation signals. Thus, we compare what happens to AB in the WT condition where A0 = B0 (by virtue of ideal dosage balance) to extreme conditions in which the dose of either A or B is increased (1.5x as in an ideal case of a heterozygous gene duplication) or halved (heterozygous deletion). We can also envisage an alternative to protein degradation by supposing that both A and B have a preferential conformation to interact with each other (*i.e.* A^I^ and B^I^ in Fig. 2b). Indeed, the 3D structure of an isolated monomer can differ from its conformation when it is complexed with binding partners forming an oligomer [18]. This is epitomized by the interactions between domains of the cAMP response element binding protein (CREB-binding protein or CBP) and the activator for thyroid hormone and retinoid receptors (ACTRs) [19]. Both the ACTR and the CBP domains are intrinsically unfolded in isolation but their co-expression leads to a stoichiometric structured complex. Thus, the assembly of a complex may involve unstable intermediates (*i.e.* “conformational transition states”) that undergo stabilization as oligomerization proceeds. This process has also been termed “interdependent protein dance” [20, 21]. If there is a stoichiometric imbalance, the monomer in excess may have time to transit for a preferred-for-binding to a nonpreferred conformation (inactive for binding). Thus, the steps A^I^ - > A^II^ and B^I^ - > B^II^ in Fig. 2b are formally equivalent to their degradation.

In our biochemical simulations, we consider for simplicity that degradation is linear and that the specific degradation rates (D) are identical. As shown in Fig. 2c, an interesting phenomenon appears when the initial concentration of either A0 or B0 is halved. In that case, the degree of buffering reaches a maximum level, when D/kAB (where kAB is the specific dimerization rate) is of the order of A0/5. In such conditions, halving the amount of A0 or B0 leads to a residual amount of 58 % of AB instead of 50 %. This point corresponds to a degree of overexpression of 123 % of AB for a 150 % increase of either A0 or B0, showing again that the buffer can act in both directions. This relationship is remarkably similar to the one mentioned above, for equilibrium conditions (*i.e.* the maximum buffering for a deletion appears at Kdissociation = A0/4) even if the mechanistic bases are totally different. Again, the existence of the buffer effect implies the existence of some excess of monomers. In the equilibrium model the buffer effect appear at the expense of the existence of uncomplexed mononomers, in the irreversible conditions, it relies on monomer degradation. According to the conformational inactivation scenario outlined above, when B0 is halved, at each time-point there will be an excess of A that can reach the inactive-for-binding conformation A^II^.

The passive buffering effect outlined here relies on the existence of degradation or conformational inactivation of the monomers in competition with dimerization. The importance of the degradation/inactivation reactions can be better appreciated in an asymmetric model, where one monomer is degradable and the other is not. As shown in Fig. 2d, in such conditions, changes of B0 (the nondegradable entity) are much better buffered than changes in A0 and, of course, in the absence of degradation of both there is no buffering at all. This asymmetric example also shows that even in the simplest case of dimers each monomer can have different dosage sensitivities (depending on the parameter settings of the system). From the perspective of the conformational inactivation scenario, monomer B can be considered as a rapid and autonomous folder (*i.e.* it reaches alone its final conformation). Because it provides information for the folding of A, its insufficiency will lead to the inactive conformation A^II^.

### The buffer effect in realistic conditions

**Fig. 3:**
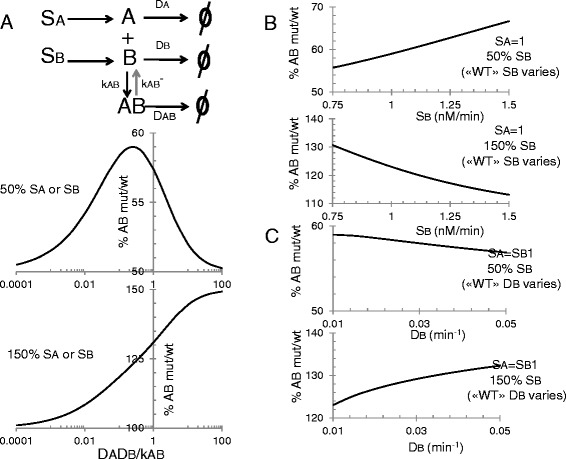
Buffer effect in the steady-state. **a** Realistic model in which synthesis of both A and B is considered along with their degradation and that of the dimer (upper panel). The lower panels represent the buffering response of heterodimer AB formation to changing the parameter DADB/k_AB_. For the “normal” conditions, the parameters were: S_A_ = S_B_ = 1nM/min, D_A_ = D_B_ = 0.01 min^−1^ and k_AB_ ranged from 0.000001 to 1. Here k_AB_^−^ = 0 because the assembly was considered to be irreversible. The ordinates represent the % of AB when either S_A_ or S_B_ is changed (0.5X or 1.5X) with respect to S_A_ = S_B_. The results were obtained using equation 1. In such conditions when MPC = D_A_D_B_/k_AB_ ranges from S_A_/5 to S_A_/4 there is maximum buffering for deletions. As discussed in the text, the findings obtained here hold for a reversible situation (only the mathematical expression of the MPC changes). **b** Buffering response of the heterodimer AB formation to changing S_B_ (as if it varied in a population). As above, S_A_ = 1 and the ratio D_A_D_B_/k_AB_ = 0.25. The span of the S_B_ values induces a (small) variation of at most 25 % of AB production in any direction with respect to S_A_ = S_B_ = 1nM/min. **c** Buffering response of heterodimer AB formation to changing D_B_ (as if it varied in a population). As above, k_AB_ = 0.0004 and D_A_ = 0.01. S_A_ = S_B_ = 1nM/min. The span of the D_B_ values displayed induces a maximum decrease of 25 % of AB production with respect to D_A_ = D_B_ = 0.01 min^−1^

In order to explore this question, the synthesis of each monomer will be represented by the fluxes S_A_ and S_B_. Their degradation will be considered again as a linear process governed by the rate constants D_A_, D_B_ and D_AB_. As above, properly speaking, S and D contain information on mRNA/protein production and degradation, respectively. Because we want to focus on what happens at the protein level only, we must assume that the processes of translation and degradation of the mRNA encoding A and B are strictly proportional to their concentrations. Thus, gene deletions or duplications can be easily modeled by changing S or D (Fig. 3a). For simplicity, we will focus on the steady-state. By using numerical simulations, as performed in the previous section, it is difficult to detect the parameter(s) modulating the buffering capacity in the steady state (if the buffer exists at all). For this reason, we solved the differential equations describing the system in the steady state (*i.e.* when dA/dt = dB/dt = dAB/dt = 0). The steady-state concentration of AB is given by the following formula:

1 $$ \left[ AB\right]=\frac{1}{2{D}_{AB}}\left({S}_A+{S}_B+\frac{D_A{D}_B}{k_{AB}}-\sqrt{{\left({S}_A+{S}_B+\frac{D_A{D}_B}{k_{AB}}\right)}^2-4{S}_A{S}_B}\right) $$

With this equation in hand, it is easier to grasp the candidate terms/parameters that should play a role in a potential buffer effect. Our question reduces to determining the maximum of the fraction AB2/AB1 when either S_A_ or S_B_ have been halved with respect to the WT situation. D_AB_ vanishes from this fraction (*i.e.* is not a candidate). Here, D_AB_ is simply a factor that modulates negatively the final AB output. Thus, we are left with D_A_D_B_/k_AB_. This ratio is called here the *monomer partition constant* (MPC) because it reflects the relative strength of the competing reactions: dimerization or degradation/inactivation. When normally S_A_ = S_B_ (and D_A_ = D_B_), there is a D_A_D_B_/k_AB_ value that elicits a maximum buffering capacity. At the point of maximal buffering for a deletion (59 % of AB) only 123 % of AB is produced when either S_A_ or S_B_ are increased by 150 % (Fig. 3a). The Fig. 3b, c shows that the buffer effect exists to different extents for rather important variations of S (from 0.5 to 1.5 nM/min) or D from 0.01 to 0.05 min^−1^. This means that according to the genotypes in a population some individuals can be weak or strong “bufferers” of a particular variation. For instance, according to Fig. 3b an individual for whom S_B_ is 1.5 nM/min will buffer very well changes in S_B_ but less well changes in S_A_.

The above results can be extended to a more complex situation, in which dimerization is reversible. Now, the MPC becomes:

$$ \frac{D_A{D}_B}{D_{AB}}\frac{k_{\mathrm{AB}}-+{D}_{AB}}{k_{AB}} $$

Because only the MPC changes in the reversible situation, there will also be buffering, as described above. However, now, D_AB_ will obviously play a role because it is involved in the MPC (and does not cancel out). A survey of the literature shows that the parameters used in our simulations are compatible with the existence of such buffering for some TFs (see [22–25]). However, this assertion has to be tempered by the fact that the parameters for the TFs in question have been determined *in vitro* and that the monomers and dimers can be involved in competing reactions (including nonspecific interactions with the rest of the cell).

The importance of AB degradation for the buffering effect when dimerization is reversible is illustrated by Eq. 2 that we have previously explored in another context [17],

2 $$ \left[ AB\right]={\left(\frac{D_A{D}_B.{k}_{AB}-}{k_{AB}}\right)}^{-1}{S}_A.{S}_B $$

In fact, when AB is not degradable, there is no buffer effect in the steady state. This contrasts with the fact that D_AB_ is simply a factor that modulates AB output in conditions of irreversibility. This also suggests that according to the assumptions of the model, buffering is more likely to emerge for stable degradable dimers (because for unstable dimers D_AB_ should be high to insure AB itself is also degraded and not only the component monomers). However, at least in theory, buffering can also be achieved in conditions of full reversibility.

Although not treated here, it is worth noting that for homodimers the amount of complex in the case of a heterozygous deletion can range from 25 % of the normal amount (as predicted in [17, 26]) to 50 % when MPC values are high. In the former case there is an enhancement of the deletion effect, whereas a strong MPC elicits a linear decrease of homodimer output, which can be considered as a buffered condition with regard to the worst situation. This behavior also applies to higher-order complexes having several copies of the same monomer as the trimer of Fig. 1. This means that the kinetics of monomer and multimer production and of their degradation modulate dosage sensibility along with the topology of the complex (as shown in Fig. 1).

### Potential significance of buffering for transcription factors

**Fig. 4:**
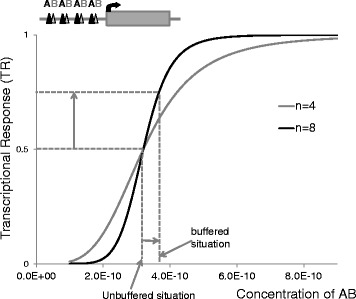
Potential impact of buffering of a deletion of an allele encoding a monomer of a heterodimeric transcription factor AB. Upper panel: Schematic representation of a target promoter with 4 binding sites for AB. Lower panel: Graph of the transcriptional response (TR) as a function of the concentration of AB. The “unbuffered situation” was conveniently chosen to elicit a TR = 0.5. The effect of buffering (57 % of AB instead of 50 %) leads to TR = 0.64 when n = 4 and 0.76 when n = 8. Thanks to the strong nonlinearity of the sigmoid, the 7 % of buffering is amplified at the level of TR. The following formula allows the calculation of TR in buffered conditions when the nonbuffered situation elicits a *TRunbuffured.* $\sqrt[n]{\frac{TRbuffered}{1- TRbuffered}\frac{1- TRunbuffered}{TRunbuffered}}=\frac{\% buffering}{50\%}$

We can now explore what the net effect of buffering would be if AB were a TF eliciting a strongly nonlinear transcriptional output. The process of recognition of a promoter P with n binding sites for AB can be represented by the global reaction P + n(AB) = P(AB)n. If we assume that the saturation fraction of the promoters P(AB)n/PT is proportional to transcription, then the normalized transcriptional response TR can be approximated as the following Hill equation:

3 $$ TR=\frac{{\left[ AB\right]}^n}{K^n+{\left[ AB\right]}^n} $$

where K and n are constants (see further explanations on the assumptions in [27]). The exponent is related to the number of binding sites per promoter and they have been equated here for simplicity. The higher n is, the steeper will be the sigmoid. For steep sigmoidal responses, the extra 8–9 % provided by the buffering of an allele deletion can make the difference between an abnormal and a normal phenotype. Figure 4 shows how the effect of buffering of a deletion with respect to an unbuffered situation that elicits a TR = 0.5. In this case a residual amount of 57 % of AB translates into TR = 0.64 when n = 4 and 0.76 when n = 8. This buffering effect can in principle be enhanced by an extra layer of buffering provided by the fact that a TF can recognize, in addition to its binding sites in *cis*-regulatory regions, other more abundant sites throughout the accessible chromatin (not leading to transcription, *i.e.* nonfunctional binding) [28]. Indeed, models considering the existence of binding to such sites show that the concentration of functionally-bound TF (*i.e.* to *cis*-regulatory regions) changes by a factor <2X when the TF concentration is either halved or doubled [29].

## Conclusions

As mentioned above, tight co-regulation has been shown for subunits of macromolecular complexes. It should be noted that for this co-regulation to be selected for, buffering cannot occur to a complete extent. This is compatible with what we observe here, even when the system operates in the “best” buffering conditions. The evolutionary trace left by mutations such as heterozygous deletions or duplications will depend on their degree of dominance (h), which ranges from 0 to 1. If h = 0, the heterozygote will have fitness 1 (*i.e.* the same as the wild-type homozygote and the mutations will be recessive) and the mutated alleles will persist in the population for longer periods of time. At the other extreme, if h = 1, the fitness of the heterozygote is the same as that of the mutated homozygote and the mutations will be rapidly purged from the population. The buffering effect will more often lead to 0 < h < 1. In such conditions, even if the heterozygote has mild defects, it can pass the normal allele on to the next generation, whereas the mutated one will be purged from the population when found in homozygosity. The values of h can differ from gene to gene in the organism and for the same gene from one organism to another. For instance, it is well known, at least superficially, that mouse is more resilient to heterozygous mutations in transcription factors than human (*i.e.* the gene is HI in human but not in mouse in laboratory conditions [5, 16]). This may be related, at least in part, to different buffering settings in these organisms for the relevant genes. Our perspective supposes that selection can tune the parameters of the system to make it robust to mutations. This bears consequences on the debate of whether dominance (and multiallelic interactions) is a consequence of physiology or has been molded by natural selection [30].

Although the above discussion has focused on diploid organisms, it is obvious that mutations affecting translational tuning in prokaryotes will perturb dosage balance. Such mutations can affect, for instance, the Shine-Dalgarno ribosome-binding sequences of the various cistrons within an operon or affect the coding sequences themselves by, for instance, changing a preferential codon to a non-preferred one [31].

The model sketched here has experimental support and can be further tested. For instance, N-terminal acetylation-induced protein degradation is suppressed when a subunit binds to its partners within a complex [32]. Proteomic studies of aneuploid yeast and mammalian cells show that the components of protein complexes encoded on the supernumerary chromosome(s) are preferentially buffered at the protein level [33–36]. In particular, studies with yeast strains containing an extra chromosome per haploid genome showed that in the absence of transcriptional buffering the subunits of macromolecular complexes, including all ribosomal subunits detected underwent substantial passive buffering (or attenuation). Interestingly, inhibition of protein degradation led to an increase of the levels of the proteins encoded by the duplicated genes more than the rest of the genome and this was particularly critical for the components of complexes [37]. Despite this overall agreement in situations of overexpression of multiple genes (*i.e.* in aneuploids), molecular data on buffering is lacking for deletions. Thus, the ideal experimental test of our models should rely on assessing protein levels for cells carrying heterozygous single-gene deletions or duplications when transcriptional dosage compensation is ruled out. For deletions, thousands of heterozygous knock-outs in yeast [38] and mouse (embryonic stem) cells (https://www.komp.org/, [39]) are available for testing. However, it is difficult to propose a typical (proteome-wide) experiment because the expected results depend on an extensive knowledge of the stoichiometry as well as the topology of the complexes, which are often unknown.

## Methods

### Steady-state solution when the reaction of production of AB is irreversible

In order to obtain the steady-state solution for the reaction scheme of Fig. 3a, we solve the differential equations describing the system so that dA/dt = dB/dt = dAB/dt = 0.

4 $$ \frac{dA}{dt}={S}_A-{k}_{AB}A\cdot B-{D}_A\cdot A=0 $$

5 $$ \frac{dB}{dt}={S}_B-{k}_{AB}A\cdot B-{D}_B\cdot B=0 $$

6 $$ \frac{dAB}{dt}={k}_{AB}A\cdot B-{D}_{AB} AB=0 $$

In addition, we have that:

7 $$ {S}_A={D}_A\cdot A+{D}_{AB} AB $$

8 $$ {S}_B={D}_B\cdot B+{D}_{AB} AB $$

We solve AB from equation 6 by replacing A and B from Eqs. 7 and 8.

$$ \begin{array}{l}0={k}_{AB}\frac{S_A-{D}_{AB}. AB}{D_A}\frac{S_B-{D}_{AB}. AB}{D_B}-{D}_{AB} AB\\ {}0=\frac{k_{AB}}{D_A{D}_B}\left({S}_A-{D}_{AB}. AB\right)\left({S}_B-{D}_{AB}. AB\right)-{D}_{AB} AB\\ {}0=\frac{k_{AB}}{D_A{D}_B}\left({S}_A{S}_B-{D}_{AB}. AB.{S}_A-{D}_{AB}. AB.{S}_B+{D}_{A{B}^2}.A{B}^2\right)-{D}_{AB} AB\\ {}\mathrm{M}\mathrm{ultiplying}\ \mathrm{b}\mathrm{y}\ \mathrm{the}\ \mathrm{M}\mathrm{P}\mathrm{C}\ \left(\mathrm{a}\mathrm{s}\ \mathrm{defined}\ \mathrm{i}\mathrm{n}\ \mathrm{the}\ \mathrm{main}\ \mathrm{text},\ \mathrm{i}.\mathrm{e}.\ \frac{D_A{D}_B}{k_{AB}}\right)\\ {}0={S}_A{S}_B-{D}_{AB}. AB\left({S}_A+{S}_B\right)+{D}_{A{B}^2}.A{B}^2-\frac{D_A{D}_B}{k_{AB}}{D}_{AB} AB\\ {}0={D}_{A{B}^2}.A{B}^2-{D}_{AB}. AB\left({S}_A+{S}_B+\frac{D_A{D}_B}{k_{AB}}\right)+{S}_A{S}_B\\ {}0=A{B}^2-\frac{1}{D_{AB}}\left({S}_A+{S}_B+\frac{D_A{D}_B}{k_{AB}}\right) AB+\frac{1}{D_{A{B}^2}}{S}_A{S}_B\end{array} $$

One of the solutions of this quadratic equation gives the steady-state value of AB:

$$ A{B}_{ss}=\frac{1}{2{D}_{AB}}\left(S{}_A+{S}_B+MPC-\sqrt{{\left({S}_A+{S}_B+MPC\right)}^2-4{S}_A{S}_B}\right)\mathrm{E}\mathrm{q}.\ 1 $$

### Steady-state solution when the reaction of production of AB is reversible

The set of differential equations changes to incorporate the term of AB dissociation (whose specific rate constant is represented by kAB^−^):

9 $$ \frac{dA}{dt}={S}_A-{k}_{AB}A.B+{k}_{A{B}^{-}} AB-{D}_A.A=0 $$

10 $$ \frac{dB}{dt}={S}_B-{k}_{AB}A.B+{k}_{A{B}^{-}} AB-{D}_B.B=0 $$

11 $$ \frac{dAB}{dt}={k}_{AB}A.B-{k}_{A{B}^{-}} AB-{D}_{AB} AB=0 $$

We solve for AB as described above.

$$ \begin{array}{l}0=\frac{k_{AB}}{D_A{D}_B}\left({S}_A-{D}_{AB}. AB\right)\left({S}_B-{D}_{AB}. AB\right)-\left({k}_{A{B}^{-}}+{D}_{AB}\right) AB\\ {}0={S}_A{S}_B-{D}_{AB}. AB.{S}_A-{D}_{AB}. AB.{S}_B+{D}_{A{B}^2}.A{B}^2-\frac{\left({k}_{A{B}^{-}}+{D}_{AB}\right){D}_A{D}_B}{k_{AB}}. AB\\ {}0={D}_{A{B}^2}.A{B}^2- AB\left({D}_{AB}{S}_A+{D}_{AB}{S}_B+\frac{\left({k}_{A{B}^{-}}+{D}_{AB}\right){D}_A{D}_B}{k_{AB}}\right)+{S}_A{S}_B\\ {}0=A{B}^2-\frac{AB}{D_{AB}}\left({S}_A+{S}_B+\frac{\left({k}_{A{B}^{-}}+{D}_{AB}\right){D}_A{D}_B}{D_{AB}{k}_{AB}}\right)+\frac{S_A{S}_B}{D_{A{B}^2}}\\ {}\end{array} $$

After solving this quadratic equation we obtain the steady-state value of AB which has the same form as Eq. 6 with

12 $$ MPC=\frac{\left({k}_{A{B}^{-}}+{D}_{AB}\right){D}_A{D}_B}{D_{AB}{k}_{AB}} $$

### When the reaction of production of AB is reversible and AB is not degradable

13 $$ \frac{dA}{dt}={S}_A-{k}_{AB}A.B+{k}_{A{B}^{-}} AB-{D}_A.A=0 $$

14 $$ \frac{dB}{dt}={S}_B-{k}_{AB}A.B+{k}_{A{B}^{-}} AB-{D}_B.B=0 $$

15 $$ \frac{dAB}{dt}={k}_{AB}A.B-{k}_{A{B}^{-}} AB=0 $$

The conservation of fluxes are as follows:

16 $$ {S}_A={D}_A.A $$

17 $$ {S}_B={D}_B.B $$

We solve AB from equation 15 by replacing A and B from Eqs. 16 and 17. This is another way to derive the same result already described in ref. 15.

$$ \begin{array}{l}0={k}_{AB}\frac{S_A}{D_A}\frac{S_B}{D_B}-{k}_{AB}- AB\\ {} AB=\frac{k_{AB}}{k_{AB}-}\frac{S_A}{D_A}\frac{S_B}{D_B}\end{array} $$

## Reviewers’ comments

First of all we would like to thank the referees for their comments and suggestions that were addressed as follows:

### Reviewer’s report 1

Reviewer 1: E. Koonin

Reviewer’s comment

Mechanisms and evolution of dosage compensation are fascinating and important subjects. This paper provides very simple but, to my knowledge, new and valid models of the buffering effect. This is quite useful, especially as simple, feasible tests are proposed.

A quick comment. The discussion in the article focuses on dominance that is only relevant in diploid organisms. How does the model apply to haploids such as most prokaryotes?

Authors’ response: Although the MS focuses on situations arising in diploid organisms, the dosage balance principles hold also for prokaryotes. Indeed, a systematically analysis of macromolecular complexes from *E. coli* has shown that more than half of the relevant molecules are synthesized at levels (counted as molecules per generation) very well correlated with their stoichiometry within the complexes. This proportional synthesis out of a polycistron relies on translational tuning. Li G-W, Burkhardt D, Gross C, Weissman JS: Quantifying absolute protein synthesis rates reveals principles underlying allocation of cellular resources. *Cell* 2014, 157:624–635.

And another point. The authors indicate that balance between subunits is controlled at the levels of transcription and protein degradation. However, translational control seems to be important as well, see eg. Quax TE, Wolf YI, Koehorst JJ, Wurtzel O, van der Oost R, Ran W, Blombach F, Makarova KS, Brouns SJ, Forster AC, Wagner EG, Sorek R, Koonin EV, van der Oost J. Differential translation tunes uneven production of operon-encoded proteins. Cell Rep. 2013 Sep 12;4(5):938–44.

Authors’ response: We have included this reference and discussed it in the text.

### Reviewer’s report 2

Reviewer 2. Berend Snel

The manuscript of Veitia and Birchler presents a novel and general model for protein complex formation. Interestingly even without most other cellular processes taken into account (such as transcriptional buffering or post-transcriptional buffering) the authors show that there is already counterintuitive behavior. However I also have some reservations about this manuscript which I detail below.

1) The model does not seem to connect to any concrete or specific biological question or challenge, such as for example a growth phenotype we cannot explain or a regulatory mutation that has a less strong phenotype than expected. In the discussion the authors discuss the haploinsufficiency differences between human and mouse, but to me it is not clear whether there is any specific reason to think this is due to buffering at the protein complex formation level or other levels where buffering takes place. Similarly most of the examples in the paper are not examples in the biological sense but cartoon/schematic/idealized versions of potential biological cases.

Authors’ response: The referee is correct when he says that the model does not apply to a specific system. This is mainly due to the lack of detailed molecular knowledge and also to our aim, which is basically to attract the attention of the reader to a possible buffering effect at the protein level. However, a survey of the literature shows that the parameters used in our simulations are compatible with the existence of such buffering for some TFs (see refs [22–25]). However, as stated in the text, this has to be tempered by the fact that the parameters for the TFs in question have been determined *in vitro* and that the monomers and dimers can be involved in competing reactions (including nonspecific interactions with the rest of the cell). So the problem of lack of realistic data remains the same. Concerning the issue of the difference of dosage senstivity between human and mouse, it is mentioned en passant. We do not claim that our model is the sole explanation for that (although it might contribute).

This absence of acute biological urgency is compounded by the fact that protein complex formation/concentration does not very often (that I know off) equal phenotype. Many processes and feedback loops still exist between protein complex formation and phenotype and a simple inducible transcription loop could easily provide almost perfect buffering by simply increasing the expression of the subunits which is missing from one of the chromosomes.

Authors’ response: The referee is correct but the existence of feedback does not preclude the existence of other types of less active mechanisms as the ones hypothesized and explored here.

2) Related to the first point I also got the impression that this paper misses a lot of context. For example one by now classic nature paper argues that protein complex members are not co-regulated and that the transcriptional regulation of missing subunits would serve to regulate the entire complex (just in time complex formation). In general a lot more has happened on protein complexes and expression than the papers from more than ten years ago which are used to support the link between expression and protein complex formation, see also *e.g.*http://www.biomedcentral.com/1752-0509/2/1

Authors’ response: Indeed, a systematic analysis of macromolecular complexes from *E. coli* has shown that more than half of the relevant molecules are synthesized at levels (counted as molecules per generation) perfectly correlated with their stoichiometry within the complexes in *E. coli* and yeast. Li G-W, Burkhardt D, Gross C, Weissman JS: Quantifying absolute protein synthesis rates reveals principles underlying allocation of cellular resources. *Cell* 2014, 157:624–635.

Concering the reference http://www.biomedcentral.com/1752-0509/2/1 (*i.e.* Semple JI, Vavouri T, Lehner B. A simple principle concerning the robustness of protein complex activity to changes in gene expression. BMC Syst Biol. 2008 Jan 2;2:1. doi:10.1186/1752-0509-2-1 our point of view on this specific paper has been published in Veitia RA. On gene dosage balance in protein complexes: a comment on Semple JI, Vavouri T, Lehner B. A simple principle concerning the robustness of protein complex activity to changes in gene expression. BMC Syst Biol. 2009 Jan 30;3:16. doi:10.1186/1752-0509-3-16.

3) In some places the manuscript is slightly redundant arguing the same fact/observation in more or less the same wordings. This should be edited.

Authors’ response: We have tried to avoid redundancies.

### Reviewer’s report 3

Reviewer 3. Reviewer Csaba Pal.

I must say I found the paper rather confusing. The authors viewpoint is biased. They posit that “The GDBH (gene dosage balance hypothesis) explains genetic dominance and several of its evolutionary consequences”. This may indeed be so, but the hypothesis is not universally accepted.

Authors’ response: To avoid a lengthy dicussion on the GDBH we have simply removed the sentences in question because they are not central to the point developed here. Yet, dosage balance at the protein level is obvious as demonstrated in Li G-W, Burkhardt D, Gross C, Weissman JS: Quantifying absolute protein synthesis rates reveals principles underlying allocation of cellular resources. *Cell* 2014, 157:624–635.

Most notably, an important prediction of the theory is that both underexpression and overexpression of protein complex subunits should lower fitness.

Authors’ response: This is absolutely not the case and a discussion of the topic is provided in Veitia RA. On gene dosage balance in protein complexes: a comment on Semple JI, Vavouri T, Lehner B. A simple principle concerning the robustness of protein complex activity to changes in gene expression. BMC Syst Biol. 2009 Jan 30;3:16. doi:10.1186/1752-0509-3-16. See also Dopman EB, Hartl DL. A portrait of copy-number polymorphism in Drosophila melanogaster. Proc Natl Acad Sci U S A. 2007 Dec 11;104(50):19920–5. and many others.

As regards overexpression, the data is not clear cut, and the two best systematic studies disagree whether protein complex subunits are especially prone to gene overexpression (Sopko et al. 2006, Makanae et al. 2013). Moreover, in contrast to expectation of the GDBH, in most cases the overexpression phenotypes differ from loss-of-function phenotypes. Alternative theories may also explain many of the patterns which seem consistent with the theory. For example, the primary mechanism of haploinsufficiency (*i.e.* fitness loss of heterozygous for a loss-of-function allele) in yeast could be due to insufficient protein production, rather than due to dosage imbalance *per se* (Deutschbauer et al. 2005).

Authors’ response: Haploinsufficiency by insufficient protein production is difficult to disentangle from a balance effect because an insufficient level is needed to cause an imbalance. A comparison of haploinsufficiency in a diploid to a euploid haploid state that is normal for that trait argues for a balance at least on some level.. So there is still room for imbalances leading to non linearities (be it a the level of protein complexes or cellular networks) yet to be studied.

So, despite publishing a paper supporting GDBH (Papp et al. 2003), in the light of the new data, I think the general role of the theory in genetic dominance is far from clear, to say the least. Regretfully, this paper (and many prior reviews by the same authors) ignore all these problems.

Authors’ response: These comments reflect that there is debate in the field. The reviewer ignores tens of papers (too many to be cited here) dealing with protozoa, Drosophila and plant data lending credence to the GDBH. We also note that we still have to better understand the many complexities involved with genomic balance, which include the discussion raised in this manuscript.

The field needs more systematic and genome-scale works which directly test these central issues in yeast and other organisms. Case studies and mathematical models have probably less important role at this stage.

Authors’ response: We agree that the field needs more work but it will be difficult to understand the underlying mechanisms of dominance (or buffering) without having at least working models. This is our aim here.

I may be wrong, but I had the feeling that the current manuscript shows substantial overlap with a previously published paper by one of the authors (Veitia 2010). It was not at all clear to me what the new and distinguishing predictions of the current paper are and how could they be studied experimentally in the future.

Authors’ response: The author is incorrect. There is no substantial overlap with the paper Veitia 2010. Only the case “When the reaction of production of AB is reversible and AB is not degradable.” overlaps to make the present paper self-contained (yet the mathematical derivation is different). Our main prediction (that we feel is obvious from reading the paper) is that the effect of a heterozygous deletion/duplication of one allele encoding a complex component may be buffered that protein level. This can now be tested by proteomics.

The importance of protein degradation and/or conformational inactivation for buffering is a potentially interesting thought, but it is very superficial at the moment, and relies heavily on results of prior papers.

Authors’ response: We believe that although our paper is purely theoretical, it will point to proteolysis/inactivation as potential mechanisms fostering buffering against gene dosage changes.

All in all, I think this paper only scratched the surface of an important problem, as it lacks clear guidelines for future studies. The authors should at the very least try to explore the idea of “assessing protein levels for cells carrying heterozygous single-gene deletions or duplications when transcriptional dosage compensation is ruled out”. As it stands, it is only a vague statement.

Authors’ response: The referee is correct and it is difficult to propose a typical (proteome-wide) experiment because the expected results depend on the stoichiometry and the topology of the complex. Besides, the experimenter needs to be able to determine the amount of final complex in the WT and mutated situation, which requires not only brute force proteomics but also extensive knowledge of the biochemistry of the complex(es) in question.

### Author’s response to the second round of review

We would like to thank the referees again for their comments and suggestions that we addressed as follows:

Reviewer 1, Dr Eugene Koonin, has not returned any additional comments on your manuscript.

### Reviewer’s report 2

Reviewer 2. Berend Snel

After having read the rebuttal and the revisions, I would like to make the following points. 1) I still do not see the relevance or urgency of the model presented in this paper. Insofar as I now understand the manuscript and the rebuttals, the papers mentioned by the author themselves and by the referees, do show the relevance of the general problem - dosage effects on phenotype-, but apparently the very same papers do not bear direct relevance on the precise model presented here.

Authors’ response: The referee is right when he says there is no urgency for this model. It simply brings up a possibility of buffering that can explain why heterozygous deletions of genes encoding components of macromolecular complexes do not always lead to an abnormal phenotype (*i.e.* the wild-type allele is dominant). As already said, the idea behing this model is not to provide a quantitative appraisal of the papers mentioned in the manuscript.

2) After reading the discussion between the other referees and the authors, and some further reading I now think the model and its result are not as new as I thought in first instance. A similar model with similar results was published by one of the authors of the current paper in the Journal of Theoretical Biology in 2003. To me the current manuscript is similar to the older paper because the JTB paper also shows some degree of “free buffering” when considering statically regulated protein concentrations forming into protein dimers.

Authors’ response: The referee is right when he says that the results between the JTB 2003 paper and the present one are similar. The interesting thing is that in the first case, the basis of the buffer effect is pure “mass action”. In the present case, the important and novel element is protein degradation or conformational inactivation. We hope the reader will understand that the models are obviously different even if the results are similar.

### Reviewer’s report 3

Reviewer 3. Reviewer Csaba Pal.

I have no further comments on this manuscript. There are several key point in the authors’ response I disagree with (most particularly major predictions of the dosage balance theory), but discussing them would go beyond the scope of the manuscript.

Authors’ response: It would be interesting to know which are “the major predictions of the dosage balance theory” Dr. Pal refers to because there are many predictions in the literature said to stem from the GDBH that are either wrong or not justified. We agree with him that this discussion would be beyond the narrow scope of our theoretical paper but we would be delighted to further discuss on this matter with the referee.

## Abbreviations

ACTRs: Activator for thyroid hormone and retinoid receptors
CREB-binding protein or CBP: cAMP response element binding protein
GDBH: Gene dosage balance hypothesis
HI: Haploinsufficiency
MPC: Monomer partition constant
TF: Transcription factors
TR: Transcriptional response
WT: Wild-type
